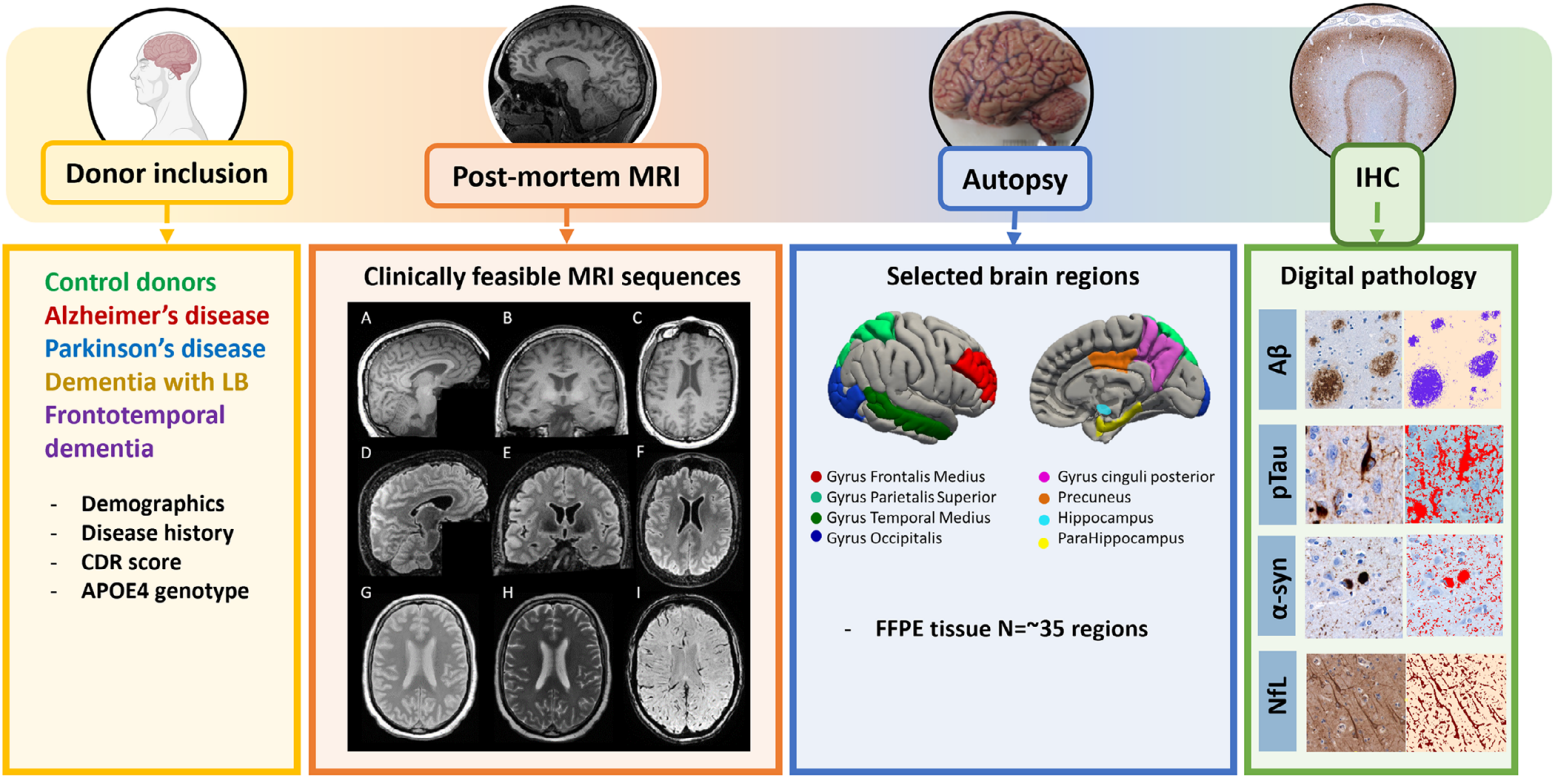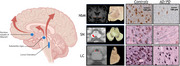# MRI measures of neuropathology in neurodegenerative disease

**DOI:** 10.1002/alz70856_098993

**Published:** 2025-12-24

**Authors:** Laura E. Jonkman, Niels Reijner, Maud M.A. Bouwman, Alex J. Wesseling, Natasja A. C. Deshayes, Chen‐Pei Lin, Irene Frigerio, Yolande A.L. Pijnenburg, Louise van der Weerd, Frederik Barkhof, Annemieke J.M. Rozemuller, Wilma D.J. Van de Berg

**Affiliations:** ^1^ Amsterdam UMC, location VUmc, Amsterdam, Netherlands; ^2^ Alzheimer Center Amsterdam, Neurology, Amsterdam UMC, Amsterdam, Netherlands; ^3^ Leiden UMC, Leiden, Zuid‐Holland, Netherlands; ^4^ Institutes of Neurology & Healthcare Engineering, University College London, London, United Kingdom; ^5^ Amsterdam UMC, location VUmc, Amsterdam, Noord‐Holland, Netherlands

## Abstract

**Background:**

Various neurodegenerative diseases fall under the umbrella term dementia: Alzheimer's disease (AD), Lewy body dementia (LBD), including Parkinson's disease dementia and dementia with Lewy bodies, and Frontotemporal dementia (FTD). Their common denominator is the pathological accumulation of misfolded proteins, affecting neuron, axon and synapse function, leading to a progressive deterioration of cognitive functioning. To define disease state, monitor disease progression and possible treatment strategies, non‐invasive and pathology‐sensitive neuroimaging biomarkers are highly needed.

**Method:**

Here we showcase several studies which define the neuropathological substrates of imaging outcome measures with an unique within‐subject correlative post‐mortem *in‐situ* MRI and neuropathology approach in >100 brain donors with neurodegenerative disease. The utilization of MRI ranges from cortical thickness and microstructural integrity to measures of connectome (dys) function. Digital quantitative pathology of Aβ, pTau, α‐synuclein, and TDP‐43 inclusions are included, as well as markers for neuroinflammation, myelin, neuro‐axonal degeneration, and synaptic density.

**Result:**

Our studies show that (ii) postmortem *in‐situ* MRI is a good proxy for antemortem *in‐vivo* MRI. (ii) cortical atrophy patterns in AD are positively associated with Aβ plaques and negatively with neuro‐axonal damage. (iii) T1w/T2w ratio is a broader indicator of cortical integrity than just myelin. (iv) LATE co‐pathology in AD and LBD has an additive effect on amygdala and hippocampal volume loss. (v) A global increase in α‐synuclein pathology has a widespread effect on brain network reorganization in LBD. (vi) Hippocampal subfields are selectively vulnerable to protein aggregations and synaptic degeneration, associated with MRI volume loss and cognitive outcome in AD and LBD. (vii) cell loss in mono‐aminergic and cholinergic nuclei in AD and LBD are associated with microstructural alterations within these regions and their cortical projections.

**Conclusion:**

By integrating multisource data (clinical, radiological, histopathological), these types of studies will result in new fundamental knowledge leading to improved interpretation of imaging datasets in the spectrum of neurodegenerative diseases.